# Current Knowledge of Breastfeeding Among Health Workers in a Developing Country Setting: A Survey in Calabar, Nigeria

**DOI:** 10.7759/cureus.10476

**Published:** 2020-09-15

**Authors:** Joanah M Ikobah, Offiong Ikpeme, Ogban Omoronyia, Nnette Ekpenyong, Ekong Udoh

**Affiliations:** 1 Hepatology and Nutrition Division, Department of Paediatrics, University of Calabar/University of Calabar Teaching Hospital, Calabar, NGA; 2 Department of Paediatrics, University of Calabar/University of Calabar Teaching Hospital, Calabar, NGA; 3 Department of Community Medicine, University of Calabar/University of Calabar Teaching Hospital, Calabar, NGA; 4 Department of Paediatrics, University of Uyo/University of Uyo Teaching Hospital, Uyo, NGA

**Keywords:** breastfeeding, health workers, knowledge, child survival, nigeria

## Abstract

Background

Health workers are in a strategic position to provide correct information to mothers on breastfeeding practice. This study assessed knowledge of breastfeeding among health workers in health facilities in Calabar.

Methods

This was a cross-sectional descriptive study. A 45-item self-administered questionnaire was used to obtain data. Ethical clearance for the study was obtained from the Cross River State Research and Ethics Committee. Data were analyzed using SPSS version 21.0 (SPSS, Inc., Chicago, USA). A knowledge score of at least 90% was considered satisfactory. Factors associated with the level of knowledge were determined using chi-square. The p-value was set at 0.05.

Result

Two hundred and twenty-five healthcare professionals were surveyed, with a mean age of 37.5 ± 9.4 years, ranging from 20 to 65 years. The commonest age group was 41 to 50 years (43.1%). Females (80.9%) formed a larger proportion of participants with a female-male ratio of 4:1. The mean percentage of knowledge score was 85.1 ± 9.0%. A satisfactory level of knowledge was found in 27.1% of respondents. About one-third (33.7%) and one-fifth (21.8%) of health workers were not aware of the weight control benefit and protection against osteoporosis of breastmilk, respectively. Approximately one-fifth (22.2%) of respondents had misconceptions concerning the effects of colostrum on the prevention of neonatal jaundice. Nurses with diploma level of training had a satisfactory level of knowledge, compared with other professions (p < 0.05).

Conclusion

Health workers’ knowledge of breastfeeding was generally good though suboptimal. Health-related professions should provide current information on the best breastfeeding practices.

## Introduction

Breastfeeding is a public health strategy for improving infant morbidity and mortality [1]. Exclusive breastfeeding decreases the risk of mortality from diarrhea and pneumonia [2]. It supports the immune system of the infant and could protect from later life non-communicable diseases such as diabetes and obesity [2]. The World Health Organization (WHO) and United Nations Children’s Fund (UNICEF) recommends that every infant be exclusively breastfed for the first six months of life, with breastfeeding continuing for up to two years of age [3]. The UNICEF 2019 global databases show that only 44% of newborns are commenced on breastmilk within one hour of delivery and about 40% of infants are exclusively breastfed in the first six months of life [2].

The exclusive breastfeeding rate for children under six months in Nigeria is 29% following the 2018 Nigeria Demographic Health Survey (NDHS) with the average duration of exclusive breastfeeding in Nigeria being 2.8 months [4]. This is the highest in Nigeria in the last decade from 2008 to 2018, where the rate was 13% in 2008 and 17% in 2013% [4,5].

There are many factors contributing to the unacceptable low rates of breastfeeding globally [6-8]. One of the factors is the role of health workers. Health workers play a key role in supporting the initiation and establishment of effective breastfeeding of neonates by their mothers. Health workers are in a strategic position and have the responsibility to educate and counsel mothers and the general public towards proper initiation and adherence to recommended infant feeding practices [6-8]. Health workers are expected to have at least sufficient knowledge of various aspects of breastfeeding, including its benefits, proper techniques, existing myths, and practical aspects of managing potential challenges. Inadequate support by health workers towards positive breastfeeding practices has been reported in the past [9,10]. Okolo and Ogbonna working in Keffi, Nigeria showed that there was poor awareness of health workers in promoting and sustaining breastfeeding [11]. In a study carried out in Ikom, South-South Nigeria, about one-third of the health workers could not name more than two components of breast milk and 75.1% failed to identify more than three advantages of breastfeeding [12]. Improved healthcare practices stand out as being the most promising means of reinforcing the prevalence and duration of breastfeeding [10]. 

The majority of nursing mothers are largely dependent on health workers for acquisition and improvement in their knowledge of breastfeeding [12]. 

Health workers are an important source of support for breastfeeding mothers and their knowledge can influence a mothers' decisions to initiate and continue breastfeeding. There is a dearth study on the knowledge of healthcare professionals on breastfeeding in Cross River State hence this study. Findings from this study will be used in developing appropriate strategies to promote breastfeeding practices in Cross River State and Nigeria as a whole.

## Materials and methods

Study area

The study was conducted in Calabar, the capital city of Cross River State, in the south-south geo-political zone of Nigeria.

Study design

This was a cross-sectional study.

Study population

The study participants comprised of nurses, medical doctors, community health workers, pharmacists, and physiotherapists. Participants were attendees at a nutrition workshop in Calabar drawn from primary health centers, private hospitals, secondary health facilities, and tertiary health facilities in Calabar.

Sample size estimation

The formula for single proportion [13] n = z2pq/d2 was used to calculate sample size in this study, where n was the sample size, “z” critical value set at 95% alpha level of significance (1.96), “d” was estimated margin of error (0.05), “p” was estimated proportion of health workers with adequate knowledge of exclusive breastfeeding (0.84) as reported in the previous study [14] and q = 1- p = 0.16. With the assumption of a 5% non-response rate, this yielded a minimum sample size of 217.

Sampling technique

Attendees were recruited to participate in the workshop and the study from their different health institutions (place of practice) in Calabar, using a systematic random sampling technique, with registers obtained from the Cross River State Ministry of Health as the respective sampling frames. Each sampling interval (k) was calculated by dividing the number of relevant staff in the different institutions (using available registers) by the proportionately allocated sample size for that institution. Hence, sampling intervals k of 5, 3, 3, 4, 2 was estimated for tertiary (622/122), general (57/19), private (127/39) hospitals, and primary health care (86/23) and other (47/22) centers, respectively. Subjects were recruited by balloting between the first participant and the kth participant on the respective registers. Thereafter, subsequent subjects were recruited using the estimated sampling interval for the different institutions. If a subject did not consent to participate, the next staff on the register was selected and sampling continued from that point. Sampling was carried out until the estimated sample size was completed. 

Data collection

A 45-item self-administered semi-structured questionnaire assessing different aspects of breastfeeding was used to obtain data from consenting participants. The questionnaire consisted of demographic data of the participants and breastfeeding knowledge: benefits to babies, benefits to mothers, colostrum, effective feeding, timing and duration of breastfeeding and complementary feeding, common breastfeeding problems, and practical aspects of breastfeeding. 

Ethical issues

Ethical clearance for the study was obtained from the Cross River State Research and Ethics Committee. The confidentiality of the participants was preserved by the use of identification numbers

Statistical analysis

Data were analyzed using Statistical Package for Social Sciences (SPSS version 21.0, Chicago, Inc., USA). The participant’s characteristics were described using frequency and percentage for categorical variables and means for continuous variables. The sum of correct responses was determined for each respondent. A knowledge score of ≥ 90% was classified as Good, 50-89% classified as Fair while < 50% was classified as Poor. The knowledge score was further classified as satisfactory if ≥90% or unsatisfactory if <90%. Pearson’s chi-square test was used to determine the association between the socio-demographic variables of the participants and their knowledge of breastfeeding. The test was deemed significant at p-value < 0.05. 

## Results

Socio-demographics of participants

Two hundred and twenty-five healthcare professionals including medical doctors, nurses, community health workers, and others were surveyed, with a mean age of 37.5 ± 9.4 years, ranging from 20 to 65 years. The commonest age group was 41 to 50 years (43.1%). Most subjects were females (80.9%), married (59.6%), nurses (60.0%), practiced in a tertiary hospital (54.2%), and have had 10 or less years of practice experience (57.8%; Table 1).

**Table 1:** Sociodemographic characteristics of respondents (N = 225)

Variable	Frequency	Percentage
Age group (years)		
<30	57	25.3
31-40	97	43.1
41-50	39	17.3
>50	32	14.2
Total	225	100
Gender		
Male	43	19.1
Female	182	80.9
Total	225	100
Marital status		
Married	134	59.6
Single	83	36.9
Widowed	6	2.7
Divorced/separated	2	0.9
Total	225	100
Highest professional qualification		
Diploma	134	59.6
First degree	63	28
Certificate	22	9.8
Second degree	6	2.7
Total	225	100
Place of practice		
Tertiary hospital	122	54.2
Private hospital	39	17.3
PHC	23	10.2
General hospital	19	8.4
Others	22	9.8
Total	225	100
Duration of practice (in years)		
10 or less	130	57.8
11-20	51	22.7
21-30	34	15.1
>30	10	4.4
Total	225	100

Table 2 shows frequency distribution of correct and incorrect responses of participants to the knowledge of benefits and principles of breastfeeding. Most subjects knew the benefits of breastfeeding to babies and mothers, except for knowledge items 9 and 11, where approximately one-third (33.7%) and one-fifth (21.8%) did not know of the weight control benefit and protection against osteoporosis, respectively. Approximately one-fifth (22.2%) of respondents had misconception concerning effects of colostrum on prevention of neonatal jaundice (item 16).

**Table 2:** Frequency distribution of knowledge of breastfeeding benefits and principles (N = 225)

S/N	Breastfeeding Knowledge Item	Correct	Incorrect
		n (%)	n (%)
Benefits to babies			
1	Breastfeeding reduces the risk of respiratory infection among babies	219 (97.3)	6 (2.7)
2	Breastfeeding increases the baby’s intelligence	216 (96.0)	9 (4.0)
3	Breastfeeding helps to reduce the incidence of child abuse and neglect	219 (97.3)	6 (2.7)
4	Baby who received breastfeeding is less prone to get diarrhea	215 (95.6)	10 (4.4)
5	Breast milk provides baby with more protection from allergy compared to formula milk	221 (98.2)	4 (1.8)
6	Breastfeeding causes good development of baby’s teeth and gum	211 (93.8)	14 (6.2)
Benefits to mothers			
7	Exclusive breastfeeding is beneficial in spacing birth	217 (96.4)	8 (3.6)
8	Breastfeeding helps to stimulate uterine contraction	214 (95.1)	11 (4.9)
9	Mothers who practiced breastfeeding may achieve pre-pregnancy weight faster	138 (61.3)	87 (33.7)
10	Frequent breastfeeding may prevent breast engorgement	201 (89.3)	24 (10.7)
11	Mother who practiced breastfeeding has a low risk of getting breast cancer	212 (94.2)	13 (5.8)
12	Breastfeeding may protect against osteoporosis	176 (78.2)	49 (21.8)
Colostrum			
13	Colostrum is the mother’s early milk, which is thick, sticky, and yellowish	216 (96.0)	9 (4.0)
14	Colostrum is difficult to digest and needs to be discarded	217 (96.4)	8 (3.6)
15	Colostrum causes constipation among babies	211 (93.8)	14 (6.2)
16	Colostrum is not able to protect babies from jaundice	175 (77.8)	50 (22.2)
Effective feeding			
17	Babies will gain weight if they receive effective feeding	210 (93.3)	15 (6.7)
18	Correct positioning helps to achieve effective breastfeeding	221 (98.2)	4 (1.8)
19	Babies sleep well after they receive adequate breastfeeding	223 (99.1)	2 (0.9)
Timing and duration of breastfeeding and complementary feeding			
20	Breastfeeding should be initiated within 30 minutes after delivery	216 (96.0)	9 (4.0)
21	Breastfeeding should be given on demand	207 (92.0)	18 (8.0)
22	Baby should be allowed to breastfeed for at least 10–20 minutes per session	210 (93.3)	15 (6.7)
23	Breastfeeding should be continued up to two years even though the baby has received complementary food	210 (93.3)	15 (6.7)
24	Complementary feeding should be introduced at six months of age	215 (95.6)	10 (4.4)
25	Mothers may mix breastfeeding and formula feeding once the baby starts taking complementary food	201 (89.3)	24 (10.7)

Table 3 shows frequency distribution of correct responses to knowledge of problems and practical aspects of breastfeeding. Incorrect responses were provided by 51.1%, 24.0%, 24.9%, and 36.0% of respondents concerning the effects of inverted nipple (item 27), appropriate response to cracked nipple (item 28), and breast engorgement (items 30 and 31), respectively. Commonly misconceived practical aspects of breastfeeding included breast milk freeze storage (item 39, 67.1%), effect of massage during breast engorgement (item 33, 37.3%), regularity of breast milk expression (item 38, 33.8%), and refrigeration of breast milk (item 40, 32.0%).

**Table 3:** Frequency distribution of knowledge of breastfeeding problems and practice (N = 225)

S/N	Breastfeeding Knowledge Item	Correct	Incorrect
		n (%)	n (%)
Common breastfeeding problems			
26	Breast milk production is influenced by breast size	202 (89.8)	23 (10.2)
27	Mothers with inverted nipples cannot breastfeed their babies	110 (48.9)	115 (51.1)
28	Breastfeeding must be discontinued if the mother has a cracked nipple	171 (76.0)	54 (24.0)
29	Breastfeeding must be discontinued if the baby has jaundice	198 (88.0)	27 (12.0)
30	Breastfeeding must be discontinued if the mother has breast engorgement	169 (75.1)	56 (24.9)
31	Breast engorgement may be reduced with cold packs	144 (64.0)	81 (36.0)
Practical aspects			
32	Exclusive breastfeeding must be practiced until the infant is 6 months old	213 (94.7)	12 (5.3)
33	Massage may reduce breast engorgement	141 (62.7)	84 (37.3)
34	Giving water to the baby is encouraged after every breastfeeding	197 (87.6)	28 (12.4)
35	Belching after feeding shows that the baby is full	167 (74.2)	58 (25.8)
36	Babies who get enough feeding will pass urine more frequently	177 (78.7)	48 (21.3)
37	Oral thrush frequently happens to babies who breastfeed	176 (78.2)	49 (21.8)
38	Breast milk expression may be done every three hours	149 (66.2)	76 (33.8)
39	Expressed breast milk may be stored for three months in a freezer	74 (32.9)	151 (67.1)
40	Expressed breast milk may be stored for 24-48 hours in a refrigerator	153 (68.0)	72 (32.0)
41	It is necessary to express breast milk from one side of the breast only	207 (92.0)	18 (8.0)
42	Expressed breast milk may be mixed with previously expressed milk	183 (81.3)	42 (18.7)
43	Expressed breast milk may be warmed on a fire	194 (86.2)	31 (13.8)
44	Expressed breast milk may be warmed in a microwave	182 (80.9)	43 (19.1)
45	The leftover expressed breast milk that has been used may be stored again	164 (72.9)	61 (27.1)

Overall knowledge score of participants on breastfeeding practice

The mean score on knowledge of breastfeeding was 40.0 ± 4.2, ranging from 13.0% to 45.0%. This corresponded to mean percentage score of 85.1 ± 9.0%, ranging from 27.7% to 100%.

Table 4 shows frequency distribution of categories of level of knowledge of breastfeeding determined from the percentage knowledge scores. Most respondents 163 (72.5%) had not satisfactory (fair) knowledge of breastfeeding practice while 61(27.1%) had satisfactory (good) knowledge of breastfeeding.

**Table 4:** Frequency distribution of knowledge of breastfeeding categories (N = 225) Not satisfactory = sum of poor and fair knowledge. Satisfactory = good knowledge.

Variable	Frequency	Percentage
Sub-categories of knowledge level		
Poor (<50)	1	0.4
Fair (50-89)	163	72.5
Satisfactory (>90)	61	27.1
Total	225	100
Main-categories of knowledge level		
Not satisfactory (<90)	164	72.9
Satisfactory (>90)	61	27.1
Total	225	100

Table 5 shows relationship between socio-demographic and occupational factors and level of knowledge of breastfeeding. Significantly higher proportion of nurses with diploma level of training had satisfactory level of knowledge, compared with other professions (p < 0.05). Other variables were not found to significantly influence level of knowledge of breastfeeding (p > 0.05).

**Table 5:** Factors associated with knowledge of breastfeeding category (N = 225)

Variable	Breastfeeding Knowledge Category			Chi-Square (p-Value)
	Not satisfactory	Satisfactory	Total	
	n (%)	n (%)	n (100)	
Age groups (in years)				
<30	35 (61.4)	22 (38.6)	57 (100)	5.9
31-40	72 (74.2)	25 (25.8)	97 (100)	(0.12)
41-50	31 (79.5)	8 (20.5)	39 (100)	
>50	26 (81.2)	6 (18.8)	32 (100)	
Total	164 (72.9)	61 (27.1)	225 (100)	
Gender				
Male	29 (67.4)	14 (32.6)	43 (100)	2.8
Female	97 (53.3)	85 (46.7)	182 (100)	(0.09)
Total	126 (56.0)	99 (44.0)	225 (100)	
Level of training				
Certificate	20 (90.9)	2 (9.1)	22 (100)	Fisher's
Diploma (in nursing)	87 (64.9)	47 (35.1)	134 (100)	Exact
First degree	52 (82.5)	11 (17.5)	63 (100)	(0.01)
Second degree	5 (83.3)	1 (16.7)	6 (100)	
Total	164 (72.9)	61 (27.1)	225 (100)	
Duration of practice (years)				
<10	87 (66.9)	43 (33.3)	130 (100)	Fisher's
11-20.	39 (76.5)	12 (23.5)	51 (100)	Exact
21-30	30 (88.2)	4 (11.8)	34 (100)	(0.07)
>30	8 (80.0)	2 (20.0)	10 (100)	
Total	164 (72.9)	61 (27.1)	225 (100)	

## Discussion

The overall knowledge of health workers on breastfeeding practices in this study was 85.1 ± 9.0% which was below the cut-off of 90.0% for satisfactory knowledge. Satisfactory knowledge of breastfeeding practices was observed in 27.1% of the respondent. This is, however, lower than that observed by Utoo et al. working in the sub-urban city of Ikom [12]. The paucity of knowledge on breastfeeding practices among health workers in the index study raises concerns about the quality of health talks and counselling offered to mothers and other caregivers of young infants in health facilities.

Knowledge of health workers on the benefits of breastfeeding to babies, their mothers, colostrum, effective breastfeeding, timing, and duration of exclusive breastfeeding and complementary feeding was good. However, their knowledge was suboptimal regarding the effect of breastfeeding on the achievement of pre-pregnancy weight, protection against osteoporosis, and colostrum in the protection of babies from jaundice. In a survey conducted in Tanzania, 97% of health workers stated a lack of knowledge of the benefits of exclusive breastfeeding as one of the main reasons for the low breastfeeding rate [15]. A majority of the general public including nursing mothers largely depend on health workers for acquisition and improvement of their knowledge of breastfeeding [12].

Most of the respondents had satisfactory knowledge of the importance of colostrum. This is like that observed by Utoo et al., colostrum is known to have several immunologic properties that protect the baby against infections in early life [12]. Mothers are more likely to practice exclusive breastfeeding if they know the protective function of colostrum against several pathogenic gut organisms. However, 77.8% of health workers had poor knowledge about colostrum protecting babies from jaundice. This level of knowledge is highly unacceptable as jaundice is among the leading causes of neonatal morbidity and mortality in most developing countries [16].

About 96% of health workers knew that breastfeeding should be initiated within one hour after delivery. This finding suggests that women attended by these health workers were more likely to commence breastfeeding of their infant soon after delivery. This finding is similar to that obtained in a study among health workers in rural South Africa, which reported a knowledge rate of 96% among professional nurses [10], however, the study in Keffi, Nigeria reported a lower knowledge rate of 20% of initiation of breastfeeding within one hour after delivery [11].

Most of the respondents knew exclusive breastfeeding to be recommended for the first six months of life. This finding is similar to that obtained in Ikom, Nigeria among health workers [12]. This response of health workers if sustained and practiced by nursing mothers could improve the low exclusive breastfeeding rate in Nigeria.

Although, 12.4% of health workers believed that infants less than six months should be given water during exclusive breastfeeding. This is however lower than 19.2% and 15.7% that subscribed to the use of water alongside breastfeeding before six months of life by Okolo and Ogbonna [11] in Keffi, Nigeria and Sadoh et al. [17] among medical women in Benin City, Nigeria, respectively. This response by health workers may reflect the common cultural myth in Nigeria that breastfed babies are usually thirsty and would need to drink some water in order to be satisfied. Sachdev et al. [18] had demonstrated that fully breastfed babies in the tropics do not need water even in extremely hot dry climates. 

Knowledge concerning common breastfeeding problems was generally not satisfactory. Good knowledge was reported in 10% of the questions used to assess the participants in this domain. About a quarter of the respondents believed that breastfeeding should be discontinued if mothers have cracked nipples or breast engorgement while about half of them believed mothers with inverted nipples cannot breastfeed their babies. The above-mentioned problems for which the health workers permitted discontinuation of breastfeeding are remediable challenges that should not obviate the practice of breastfeeding. Reasons similar to the findings in this study were attributed to the premature discontinuation of breastfeeding in Keffi, Nigeria [11] and by Chale et al. [15] in Tanzania, East Africa. This may suggest that health workers in these settings will not promote breastfeeding among mothers with those conditions. Borbala [6] and Robertson [8] have shown that poor knowledge of breastfeeding among health workers impair effective breastfeeding practices.

The age of the health workers, gender, and years of practice were not significantly associated with satisfactory knowledge of breastfeeding in this study. Chale et al. in Tanzania [15] demonstrated that years of practice, age, and gender were not significantly associated with good knowledge of breastfeeding among health workers.

There was a significant association between the cadre of health staff and knowledge of breastfeeding in this study. The knowledge of breastfeeding practice was significantly higher among those with a diploma in nursing compared with other health professionals. Medical doctors had inadequate knowledge of breastfeeding compared to nurses. The observation in this study is quite understandable since most of the health education to caregivers in health facilities is performed by nurses. The finding in this study is however contrary to the report of Chale et al. [15] and Okolo and Ogbonna [11] who reported better knowledge of breastfeeding practices among medical doctors and a higher likelihood of clinicians encouraging the practice of exclusive breastfeeding compared to other health workers.

A limitation of this study is the skewness in favour of nurses in the selection of participants This bias in the selection of respondents might have influenced some of the findings of this study, though most of the respondents were from the tertiary health facility with a representative from more than ten states of Nigeria and the neighbouring Republic of Cameroun.

## Conclusions

The overall knowledge of health workers on breastfeeding practices was suboptimal. Health workers had good knowledge of the benefits of breastfeeding and breastfeeding practices but their knowledge of common problems of breastfeeding and practical steps on breastfeeding was quite low. Training and re-training of all health workers in the State on the importance of breastfeeding are necessary to improve their knowledge of breastfeeding practice.
